# Prevention Strategies and Management of Necrotizing Enterocolitis

**DOI:** 10.1007/s40746-024-00297-2

**Published:** 2024-06-03

**Authors:** Andrea Marian Colarelli, Maria Estefania Barbian, Patricia Wei Denning

**Affiliations:** 1 Department of Pediatrics, Emory University School of Medicine and Children’s Healthcare of Atlanta, Atlanta, GA, Georgia; 2 Department of Pediatrics, Emory University School of Medicine and Children’s Healthcare of Atlanta Emory University Division of Neonatology and Children’s Healthcare of Atlanta, Atlanta, GA, Georgia

**Keywords:** Necrotizing Enterocolitis, Human Milk, Donor Human Milk, Premature Infants

## Abstract

Necrotizing enterocolitis (NEC) is a serious intestinal disease which primarily affects preterm infants. The pathogenesis of NEC is multifactorial. Thus, it is complicated to study, prevent, and manage.

**Purpose of Review:**

The purpose of this review is to provide a comprehensive summary of recent research and provide recommendations for the prevention and management of NEC. Currently, management is supportive and non-specific and long-term outcomes for surgical NEC are poor.

**Recent Findings:**

The most important strategy to prevent NEC is to provide preterm infants with a human milk diet, minimize exposure to antibiotics and avoid medications that disturb the intestinal microbiome.

**Summary:**

Strategies to optimize the infant’s intestinal microbiome are critical, as disturbances in the intestinal microbiome composition are a major factor in the pathogenesis of this disease. Optimizing maternal health is also vital to prevent prematurity and neonatal morbidity. Ongoing research holds promise for the implementation of new diagnostic modalities, preventive strategies, and medical treatment options to improve outcomes for premature infants.

## Introduction

Necrotizing enterocolitis (NEC) is a devastating intestinal disease characterized by acute intestinal inflammation which can affect multiple organ systems, causing significant morbidity and mortality [1, 2]. NEC disproportionately affects preterm infants [3], with a mortality rate close to 25% [4]. Among our most premature infants and those who require surgery, mortality rates are over 30% [5]. Despite decades of research, long-term outcomes remain unfavorable [6]. Surviving infants are more likely to experience adverse neurodevelopmental outcomes [7] and gastrointestinal sequelae [8].

**Fig. 1:**
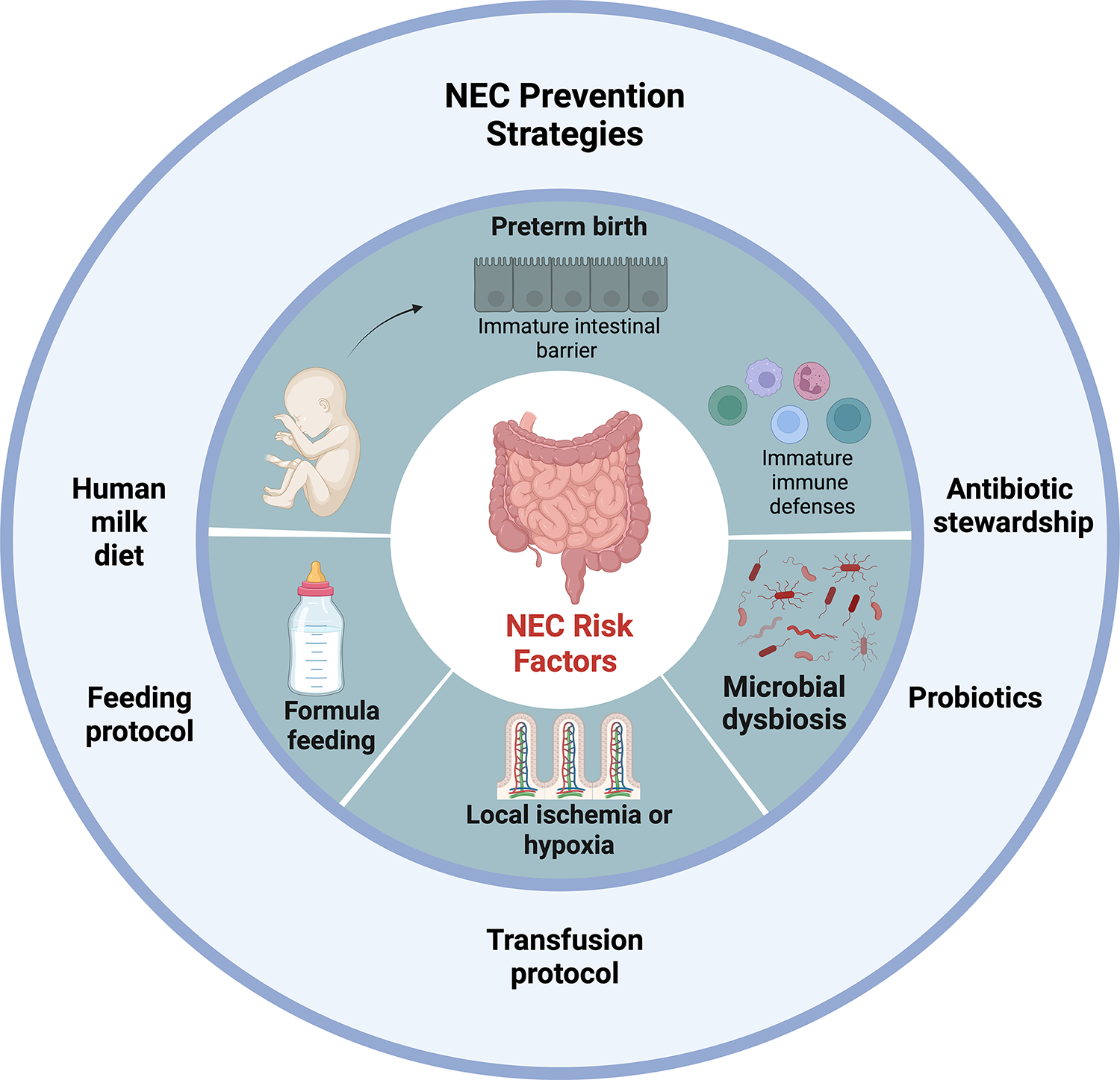
NEC Risk Factors and Prevention Strategies. NEC risk factors include preterm birth (causing immature intestinal barrier and immune defenses), formula feeding, microbial dysbiosis and local ischemia or hypoxia. NEC prevention strategies include human milk diet, implementation of feeding protocols, transfusion protocols, probiotics and antibiotic stewardship

The pathophysiology of NEC is complex and involves many dynamic factors including feeding, intestinal immaturity, microbial dysbiosis, immature immune defenses and local ischemia or hypoxia (Fig. 1) [3, 9]. These factors contribute to a breakdown and dysfunction of the intestinal barrier which can lead to bacterial translocation, excessive inflammation, and intestinal necrosis [9]. Interventions focused on preventing this cascade serve as our primary vehicle in caring for premature infants with NEC. Our goal in this review is to describe the current prevention and management strategies and future directions in NEC research.

## Prevention Strategies

### Human Milk

Human milk (HM) is the optimal infant nutrition, and the AAP recommends its use in term and preterm infants [10]. HM contains macro and micronutrients essential for infant growth and bioactive factors which promote gastrointestinal development and protect against infection and inflammation [11, 12]. These bioactive factors include commensal bacteria, HM oligosaccharides, immunoglobulins, lactoferrin, and dietary polyunsaturated fatty acids [13]. HM reduces the risk of NEC in a dose dependent fashion [14]. Initiating and sustaining lactation is often challenging for mothers with infants in the Neonatal Intensive Care Unit (NICU).

### Donor Human Milk (DHM)

DHM should be used in preterm infants when mother’s own milk (MOM) is unavailable or contraindicated. DHM diet reduces the risk of NEC compared to formula diet in preterm infants but may not confer the other benefits of MOM such as reduction in BPD, ROP, sepsis, and neurodevelopmental benefits [15]. DHM is often composed of human milk donated from mothers of term infants [16, 17], which has lower macro and micronutrients than preterm MOM [18]. Additionally, while pasteurization of DHM is necessary for safety, pasteurization and milk banking reduces the milk’s fat content, immune factors, and enzymes [17, 18].

### Human Milk and NEC

HM reduces the risk of NEC. In a recent systematic review and meta-analysis of 6 RCTs with 1,626 infants, the authors found that MOM or DHM compared to a formula diet reduced the risk of NEC (RR = 0.62, 95% CI 0.42–0.93) [19].

Using the same comparison, authors found a similar risk reduction in NEC when evaluating 18 observational studies with 6,405 infants (RR 0.45, 95% CI 0.32–0.62). These results parallel those of a Cochrane review [20]of 11 RCTs with over 1,800 preterm or low birthweight (LBW) infants and found a nearly two-fold higher risk of developing NEC in formula versus DHM-fed infants (RR 1.87, 95% CI 1.23–2.85) [20]. Moreover, the authors estimated one additional case of NEC for every 33 infants who received formula.

### Fortification of Human Milk

While HM is the gold standard diet for infants, preterm infants require higher calories, protein, minerals, and electrolytes than term infants [14]. Therefore, HM fortification is necessary for adequate growth and development [21]. HM can be fortified with bovine-milk based fortifier, formula, or DHM-based fortifier. While HM diet clearly reduces NEC, studies do not support an additional protective benefit of DHM-based fortifier over bovine-milk based fortifier. Quigley et al. found HM was protective against NEC even with bovine-milk based fortifier compared to an exclusive formula diet [16]. Two RCTs compared an exclusive HM diet (DHM + HM-based fortifier) to a diet with formula if MOM was not available (MOM + bovine milk-based fortifier or preterm formula if no MOM) and showed decreased rates of NEC in the exclusive HM diet [22, 23]. One RCT compared a HM diet with DHM fortifier to a HM diet with bovine-milk based fortifier [24] found no differences in feeding intolerance or intestinal inflammation assessed by fecal calprotectin. Both groups had the same number of NEC cases, but this study was underpowered to detect a difference in NEC [24]. In a multicenter randomized controlled trial, Jensen et al. evaluated the effect of HM diet with DHM fortification versus HM diet with bovine milk fortification on a composite outcome of NEC (stage II-III), culture positive sepsis or death among extremely preterm infants. The authors of this study report that DHM-based fortification did not reduce the primary outcome compared to bovine-milk based fortification [25•]. Thus, current evidence demonstrates that there is no added benefit of DHM-based over bovine-milk based fortifier on NEC prevention.

### Promoting MOM

Provision of MOM is the most effective strategy to reduce NEC [26, 27]. The following are strategies which can improve provision of MOM [28•]:

- Pumping/hand expression ideally within 1 hour of delivery [29, 30]
- Pumping every 3 hours
- Oral colostrum care
- Promoting skin-to-skin contact in NICU [31]
- Lactation support through licensed lactation consultant
- Sharing importance of MOM with parents and support people

## Feeding Protocols

Implementation of a standard feeding protocol (SFP) benefits infant growth and decreases NEC incidence [8, 32–35]. A Cochrane review analyzed 6 observational studies from 1978–2003 before and after SFP implementation. Random effects model revealed SFP implementation reduced the incidence of NEC by 87% [34]. Several observational studies have confirmed these results in VLBW infants [32, 33, 35]. Similar evidence exists for ELBW infants. Shah et al. found the odds of NEC were lowered by 63% in ELBW infants after SFP implementation (OR 0.38, 95% CI 0.142–0.993) [8]. Notably, these studies implemented SFPs that differed in duration of trophic feeds, rate of advancement of feeds, and timing of fortification. Therefore, a unit’s adherence to a protocol is critical to reduce NEC. A SFP should address 1) initiation and duration of trophic feeds, 2) prioritization of HM and 3) feeding advancement rate and timing of fortification [8].

### Time to Initiate Feeds

Studies have evaluated the optimal SFP design, specifically timing of feed initiation and advancement rate. A recent meta-analysis compared 14 RCTs that implemented early (< 72 h of life) or delayed (≥ 72 h of life) enteral feeds in preterm or LBW infants [36•]. Early initiation of enteral feeds had no effect on NEC risk (RR 1.05, 95% CI 0.75–1.46). Similarly, a 2022 Cochrane review evaluated timing of enteral feed initiation in VLBW infants with early (< 4 days of life) or delayed (≥ 4 days of life) feeds and found no effect on NEC risk [37•]. However, delayed enteral feeds may increase the risk of invasive infections (RR 1.44, 95% CI 1.15–1.80).

### Feeding Advancement

Historically, older studies suggested an association between feed advancement rate and risk of NEC [38, 39]. However, newer evidence from the SIFT trial did not confirm this association [40]. The SIFT trial compared fast (30 ml/kg/day) versus slow (18 ml/kg/day) enteral feed advancement rates in very preterm or VLBW infants and found that advancement rate did not affect NEC risk (aRR 0.88, 95% CI 0.68–1.16). These findings are supported by a recent Cochrane review that concluded that slow (15–24 ml/kg/day) enteral feed advancement rates did not reduce NEC risk when compared to fast (30–40 ml/kg/day) feeding advancement in VLBW infants [41•]. Conversely, a single-center cohort study (using historical controls) of infants < 750 g at birth found a reduced incidence of NEC and death after implementing slow advancement protocol (but higher rates of cholestasis) [42]. This result may due to the implementation of a SFP that didn’t previously exist or indicate that micropremies (< 750 g or < 25 weeks’ gestation) are a unique population [43]. Preterm growth restricted infants and infants with a history of absent or reverse end diastolic flow may also be at increased risk for NEC and TPN-associated liver injury [44–47]. In these populations, initiating small-volume enteral feeds of MOM early may promote growth, prevent TPN-associated liver injury and reduce the risk of NEC [45].

## Probiotics

Probiotic use for NEC prevention has been thoroughly investigated through preclinical and clinical studies [48]. Pre-clinical studies from various animal models show that probiotic supplementation reduces the risk of NEC by nearly 50% [49]. Since 2002, over 50 RCTs of over 10,000 preterm infants have been conducted to assess the effect of probiotic administration on NEC incidence [50]. There is heterogeneity in these RCTs with regards to probiotic used, gestational age/birthweight of infants included; however, several systematic reviews and meta-analyses that have evaluated these RCTs have found that probiotic use in preterm infants reduces the risk of NEC [50]. In the United States (US), probiotics are not FDA regulated. However, in 2023, the FDA issued a warning to NICUs and providers using probiotics to reduce NEC. Following this warning, unfortunately, the use of probiotics in NICUs in the US has all but halted. Based on the extensive data on probiotics, we would recommend probiotic use as an effective tool in reducing the risk of NEC.

## Antibiotic Stewardship

Preterm infants are often exposed to empiric antibiotics upon NICU admission [51]. Despite the benefit of treating active bloodstream infections, antibiotics may affect infants’ intestinal microbiome and function, thereby influencing their risk of NEC [52, 53]. In 2009, Wang et al. evaluated the fecal microbiome composition of preterm infants with and without NEC through 16S rRNA gene sequencing. This study found that preterm infants had less diversity and an increase in *Gammaproteobacteria* just prior to NEC development when compared to preterm infants who did not develop NEC. Additionally, this study found that patients who developed NEC had longer antibiotics exposure [54]. Furthermore, in 2017 Pammi et al. performed a systematic review and meta-analyses of fecal microbiome profiles in preterm infants [55]. This study included 14 studies with 106 NEC cases, 278 controls and over 2000 fecal samples. They found that the fecal microbiome of preterm infants had increased abundance of Proteobacteria and decreased relative abundance of Firmicutes and Bacteroidetes before the onset of NEC [55].

### Observational Studies

Several observational studies suggest prolonged early, empiric antibiotic use is associated with an increased NEC risk [56–63]. In a retrospective cohort study of 4,039 ELBW infants, Cotton et al. examined associations between length of first antibiotic course and NEC [56]. The authors concluded that prolonged empiric antibiotics (≥ 5 days) were associated with an increased risk of NEC or death compared to treatment < 5 days (OR 1.30, 95% CI 1.10–1.54). Similarly, a multi-center retrospective case–control study compared 224 NEC cases with 447 matched controls and found early, empiric antibiotic treatment for ≥ 5 days was associated with increased odds of NEC (aOR 2.02, 95% CI 1.55–3.13) [60]. Another large multi-center retrospective cohort study of over 14,000 VLBW infants demonstrated that early antibiotics for > 3 days increased the risk of negative outcomes, composite outcome of mortality, or any major morbidity, including NEC) (aOR 1.24, 95% CI 1.09–1.41 and aOR 1.38, 95% CI 1.25–1.51, respectively) [64].

### Randomized Control Trials

RCTs evaluating prophylactic antibiotic use for NEC prevention are limited to older studies from the 1970s-1990s which used primarily oral antibiotics for 7–24 days [65–70]. Four of these six studies demonstrated the benefit of oral antibiotics, while the other two found no significant difference. Despite these promising results, due to concern for antibiotic resistance and microbial dysbiosis, the use of oral antibiotics to prevent NEC was not adopted. If oral antibiotics such as Gentamicin can be timed with NEC-associated changes in the infant microbiome (*Gammaproteobacter* bloom), this may be a future strategy to prevent NEC.

### Systematic Reviews and Meta-Analyses

Several systematic reviews and meta-analyses have included both older RCTs and newer observational studies. Rina et al. evaluated 2 RCTs and 11 observational studies and found initial empiric antibiotic therapy ≥ 5 days was associated with an increased NEC incidence (aOR1.51, 95% CI 1.22–1.87) [71]. Similarly, Klerk et al. analyzed 10 observational cohort studies comparing initial empiric antibiotic therapy and prolonged empiric antibiotic therapy (3–14 days) and found prolonged antibiotics were associated with an increased risk of NEC (OR 2.72, 95% CI 1.65–4.47) [72•]. A meta-analysis by Fan and colleagues including 6 RCTs and 3 observational studies also found that prolonged empiric antibiotic treatment increased NEC incidence (1.31, 95% CI 1.08–1.59) [73].

These studies suggest that long courses of empiric antibiotics (≥ 5 days) may increase the risk of NEC. Therefore, we recommend prudent use of prolonged antibiotics, especially in the case of culture negative sepsis.

### Medications to Avoid

Promoting a healthy gut microbiome in preterm infants is an important step in preventing NEC. This includes promoting breastfeeding, antibiotic stewardship and avoiding medications that may alter the microbiome such as acid suppression medications. A pooled analysis of observational studies, involving over 11,000 infants showed a significant increase in NEC with administration of acid-suppression medications (OR: 1.78, 95% CI: 1.4–2.27) [74].

## Erythropoiesis-Stimulating Agents for Prevention of NEC

Erythropoietin may be beneficial for endothelial cell barriers [75]. A meta-analysis of RCTs of erythropoiesis-simulating agents given to modify transfusion exposure in preterm infants, found that infants who received this therapy < 8 days after birth had decreased NEC incidence (RR: 0.69 95% CI: 0.52–0.91) [76].

## Anemia, Transfusions and NEC

Anemia is common in preterm infants due to iatrogenic blood loss from frequent blood draws, impaired red blood cell (RBC) production and bleeding. Because of this, preterm infants often require RBC transfusions. The relationship between NEC, anemia and RBC transfusions has been studied in preclinical and clinical studies.

### Pre-Clinical Data

In a model of anemic mouse pups, MohanKumar et al. demonstrated that RBC transfusions triggered gut injury through toll-like receptor 4 (TLR4) activation [77]. The extent of the gut injury was related to duration of RBC storage. Interestingly, when non-anemic mouse pups received RBC transfusions, there was no gut injury. The authors found a positive correlation between the number of RBC transfusions and gut injury. This implies that anemia primes the gut for inflammation and subsequent RBC transfusion(s) result(s) in a secondary inflammatory insult culminating in intestinal inflammation and injury.

### Observational Studies

Mally et al. reported a temporal relationship with NEC and RBC transfusion as well as with NEC and the degree of anemia [78]. In a meta-analysis of 12 observational studies there was an association between RBC transfusions and NEC [79]. However, many of these observational studies looked for temporal association between NEC and transfusions. This does not indicate causality or increased risk of NEC after transfusions. Since then, there have been other observational studies with mixed results. Using a matched case–control study, Sharma et al. did not identify an association between RBC transfusions and NEC [80].

### Randomized Control Trials

The Prematures in Need of Transfusion (PINT) trial was a RCT conducted to study whether restrictive versus liberal RBC transfusion practices influence death before discharge or survival with severe morbidity [81]. This study found no statistically significant difference in the primary outcome of death or neonatal morbidity. A post hoc analysis identified mild to moderate cognitive delay was reduced in the higher hemoglobin threshold group [82]. This inspired the larger transfusion of prematures (TOP) trial, which aimed to determine whether liberal transfusion practices could improve survival without neurodevelopmental impairment (NDI) in preterm infants [83]. The TOP trial did not identify a difference in the risk of death or NDI at 22–26 months corrected gestational age (CGA) [83]. Another RCT, the ETTNO trial, identified no difference in death or disability at 24 months CGA from liberal versus conservative transfusion practices [84]. In these trials, NEC was assessed as a secondary outcome and no difference in the incidence of NEC was identified between higher versus lower transfusion threshold practices. These data suggest that neither number of transfusions nor degree of anemia prior to transfusions play a role in NEC development clinically.

### Meta-Analysis

Hay et al. evaluated the quality of evidence behind transfusion-associated NEC [85]. The authors evaluated 23 observational studies and 3 RCTs which reported NEC 48 h following a RBC transfusion or any time after a transfusion. The pooled outcome of NEC occurring within 48 h of a RBC transfusion was an OR of 1.13 (95% CI 0.99–1.29). For NEC occurring any time after a RBC transfusion, the pooled OR was 1.95 (95% CI 1.6–2.38). In the pooled outcome from RCT data, the OR 0.6 (95% CI 0.3–2.21), with NEC being more frequent in the restrictive transfusion group. However, the quality of evidence was considered very low, suggesting very little confidence in these effect estimates [85]. Meta-analyses of RCTs evaluating the effect of high versus low RBC transfusion threshold strategies have not found a difference in the risk of NEC [86].

Based on these studies, we would recommend implementation of a transfusion protocol based on the infant’s age and level of respiratory support which avoids anemia below that which has been tested in RCTs. By avoiding severe anemia, outside of the range which has been tested by RCTs such as the PINT trial and TOP trial [81, 83], we may be able to decrease risk of intestinal injury.

## Docosahexaenoic acid (DHA) for Prevention of NEC

Bernabe-Garcia et al. performed a Phase II RCT with 225 who received an enteral dose of 75 mg/kg DHA for 14 days once enteral feeds were started or control (high-oleic sunflower oil) [87•]. The authors found NEC was lower in the DHA group with no cases of NEC versus 7 cases in the control group (*p* = 0.007), with RR = 0.93 (95% CI 0.88 to 0.98) [87•].

## Optimizing Maternal Health

Last, it is imperative to remember that infant health is inextricably linked to maternal health. By improving maternal health before pregnancy and during pregnancy, we will reduce the incidence of preterm birth and LBW, which are major risk factors for NEC development [88].

## Management of NEC

### Management of Medical NEC

There is little evidence to guide clinicians in the management of NEC (Table 1). The management of NEC is largely supportive and includes bowel rest, decompression of the gastrointestinal tract, treatment with broad spectrum antibiotics and monitoring for pneumoperitoneum. Generally, clinicians treat medical NEC with 7–14 days of bowel rest and broad-spectrum antibiotics that cover intestinal flora (mostly anaerobes). Length of treatment can be guided by clinical status. Prior to starting antibiotics, a sepsis evaluation should be performed with blood and urine cultures and baseline CBC with differential. Trending inflammatory markers such as CRP or IL-6 can be considered. In the setting of positive cultures, antibiotic therapy may be tailored further. Antibiotics which cover anaerobic bacteria may be considered. Culture results can further direct therapy (tailored if positive or narrowed if negative). Careful monitoring of hemodynamic status and respiratory status is important as patients with NEC may require escalation of care.

### Management of Surgical NEC

The most well-accepted indication for surgery in NEC is pneumoperitoneum from intestinal perforation [89]. Other clinical and laboratory indicators include portal venous gas, abdominal wall erythema, hypotension, metabolic acidosis, hyponatremia, neutropenia, and thrombocytopenia, which have been associated with the need for surgical intervention [90], particularly if persistent. Failed medical treatment as an indication for surgery is associated with poor outcomes, suggesting that earlier surgical intervention may be beneficial in these cases [91].

The standard surgical approach is laparotomy for excision of necrotic bowel, but its challenges in critically ill infants prompt discussion about peritoneal drain placement. A recent systematic review concluded that neither RCTs nor observational studies with high quality of reporting showed a difference in mortality when comparing laparotomy versus peritoneal drainage (pooled OR 0.85, 95% CI 0.47–1.54 and pooled OR 0.67, 95% CI 0.37–1.19, respectively) [92]. In the Necrotizing Enterocolitis Surgery Trial (NEST), the authors concluded that with a preoperative diagnosis of NEC, initial laparotomy is more likely than peritoneal drainage to reduce death or NDI, with a Bayesian posterior probability of 97%. This suggests that laparotomy, rather than peritoneal drain, may reduce death or NDI in infants with surgical NEC [93••].

In summary, the surgical approach should be guided by the patient’s condition and be a discussion between the surgeon and neonatologist. Regardless of approach, the goals of surgical intervention include: 1) early intervention to minimize contamination and sepsis, 2) removal of necrotic bowel, and 3) preservation of bowel length to prevent short bowel syndrome [89].

## On the Horizon

NEC is a complex, multifactorial disease which has been extensively studied since it was first described. NEC causes significant morbidity and mortality; therefore, continued research is needed to find new modalities for earlier diagnosis of NEC, new therapies to prevent NEC and better therapies for targeted treatment of NEC. Below we summarize exciting research that may change our management of preterm infants.

### Pre-clinical Research

#### Epidermal Growth Factor (EGF) Signaling

Good et al. demonstrated that EGF signaling in amniotic fluid diminished NEC-like injury through inhibition of TLR4 [94].

#### Human Milk OligosacchaRides (HMOs)

HMOs are being studied for their ability to protect against intestinal inflammation in animal models of NEC [95, 96].

#### NEC-on-a Chip

Investigators have developed a 3-dimensional system to study the effect of therapies on human intestinal cells [97]. This microfluidic device allows investigators to manipulate the inflammatory environment to test novel NEC therapies.

#### Bile Acids

Golden et al. demonstrated that ursodeoxycholic acid reduces intestinal injury by promoting intestinal restitution through COX-2 and EGF-signaling pathways [98].

#### Mesenchymal Stem Cell Therapy

Markel et al. demonstrated that administration of mesenchymal stem cells to animals with NEC-like injury improved survival due to reduced intestinal injury and improved mesenteric perfusion [99].

#### Interleukin-22 (IL-22)

IL-22 is a cytokine that plays a critical role in maintaining the intestinal barrier, regenerating epithelial cells, and controlling intestinal inflammation. Using a mouse model of NEC, Mihi et al. revealed that IL-22 expression is minimal in neonatal mice [100•]. Additionally, the authors found that human and murine neonates lack IL-22 production during NEC [100•]. Treatment with recombinant IL-22 reduced intestinal inflammation and enhanced epithelial regeneration in their experimental model.

### Translational Research

#### NEC Biorepository

A NEC biorepository in the US has been developed with the goal of improving our understanding of the molecular indicators of NEC through biological signatures and genetic predisposition of infants with NEC [101].

#### NEC Virome

Kaelin et al. longitudinally evaluated the gut virome of preterm infants who developed NEC versus gestational age matched infants who did not develop NEC [102•]. The authors identified reduced viral beta diversity ten days prior to NEC development. This was driven by specific viral signatures and viral-bacterial interactions suggesting that the early life gut virome may be implicated in NEC development.

### Clinical Research

#### Remote ischemic Conditioning (RIC) for Prevention of NEC

A Phase II Feasibility RCT [103] evaluating the effect of RIC on NEC is ongoing. RIC is a therapy where brief cycles of non-lethal ischemia and reperfusion to a limb results in protection from ischemic damage in distant organs. In pre-clinical models of NEC, the investigators showed that RIC reduces intestinal injury [104].

#### Bowel/Abdominal Ultrasound (BUS/AUS) for Diagnosis of NEC

Currently, NEC is diagnosed by findings on abdominal radiographs. However, these findings have low sensitivity albeit high specificity for NEC diagnosis [105]. BUS is being investigated as a tool to diagnose or identify medical treatment failures of NEC earlier [106, 107]. One systematic review and meta-analysis of observational studies found that BUS findings such as focal fluid collections, complex ascites, pneumoperitoneum, bowel wall echogenicity, bowel wall thinning or thickening, absent perfusion and dilated bowel were all associated with surgery or death from NEC [108]. Whereas findings of portal venous gas or pneumatosis intestinalis were not associated with surgery or death from NEC [108]. Cuna et al. are performing a pilot RCT to establish the feasibility and pilot the design and delivery of a diagnostic RCT of BUS for NEC diagnosis [109]. There is also an ongoing phase 3 RCT evaluating the use of contrast enhanced ultrasound to evaluate bowel perfusion in infants with NEC [110] or suspected NEC.

#### GutCheckNEC

A diagnostic tool kit, created with a cohort of over 58,000 infants, provides a weighted composite risk of developing NEC [111]. This tool kit predicts surgical NEC with an area under the curve (AUC) of 0.84 (95% CI 0.82–0.85) and death from NEC with an AUC 0.83 (95% CI 0.81–0.85) [111]. The prediction of medical NEC was not as dependable with an AUC of 0.72 (95% CI 0.70–0.74).

#### NEC-Zero Project

This tool kit provides communication when deterioration is expected, limits antibiotic duration and promotes adherence to SFP [112].

#### Antibiotic Use in Preterm Infants

The NICU Antibiotics and Outcomes Trial (NANO) is an upcoming multicenter, double blinded RCT that will evaluate empiric antibiotics after birth or placebo in infants at < = 29 weeks gestation [113]. The primary outcome is composite incidence of NEC, late-onset sepsis, or death during NICU hospitalization.

#### Withholding Feeds or Continuing Feeds Around Transfusion

The WHEAT trial is an ongoing international multicenter RCT evaluating whether holding enteral feed around the time of a transfusion in babies born less than 30 weeks’ gestation will reduce the risk of NEC (NCT05213806).

## Conclusion

NEC is a multifactorial disease which requires a multifaceted approach to prevent its development in preterm infants. The most effective way to prevent NEC after preterm birth is to provide a HM diet [26, 27], ideally MOM. By promoting use of MOM, antibiotic stewardship, probiotics, standardized feeding protocols and avoiding acid-reducing medications, we can minimize microbial dysbiosis, a major factor in the development of NEC. Transfusion protocols to reduce severe prolonged anemia that may contribute to NEC risk should also be considered. Improving maternal health is of paramount importance to reduce rates of prematurity and NEC. While not much has changed in NEC treatment, promising research is being done which may lead us to earlier diagnostic and improved preventive and treatment strategies.

## Figures and Tables

**Table 1.** Management of NEC based on bell stages

Diagnosis		Management	
**Bell Stage 1:** **Suspected disease**	**Mild systemic sigs** -bradycardia, apnea, temperature instability **Mild intestinal signs** -abdominal distention, emesis, bloody stools **Non-specific or normal abdominal radiographs**	-NPO -Gastric decompression -Sepsis evaluation -Broad spectrum antibiotics -Serial abdominal radiographs	-Depending on clinical status, consider discontinuation of antibiotics and restarting feeds after 48 hours.
**Bell Stage 2:** **Definite disease**	**Mild to moderate systemic signs** **Additional intestinal signs** -absence of bowel sounds, abdominal tenderness **Radiographic signs** -pneumatosis intestinalis and/or portal venous gas **Laboratory derangements** -metabolic acidosis, thrombocytopenia	-NPO -Gastric decompression -Sepsis evaluation -Broad spectrum antibiotics -Serial abdominal radiographs (q6h) -Support respiratory and cardiovascular function	-Treat with bowel rest and antibiotics for 7–10 days. -Consider narrowing antibiotics if able after first 48 hours.
**Bell Stage 3:** **Advanced disease**	**Severe systemic signs** -respiratory failure, hypotension **Severe intestinal signs** -abdominal discoloration, severe distention **Severe radiographic signs** -pneumoperitoneum **Severe laboratory derangements** -disseminated intravascular coagulopathy, severe respiratory and metabolic acidosis	-NPO -Gastric decompression -Sepsis evaluation -Broad spectrum antibiotics -Serial abdominal radiographs (q6h) -Support respiratory and cardiovascular function -Transfer to center with Pediatric Surgery	-Treat with peritoneal drain or laparotomy, bowel rest and antibiotics for 14 days.
